# Two di-leucine motifs regulate trafficking and function of mouse ASIC2a

**DOI:** 10.1186/s13041-016-0190-x

**Published:** 2016-01-27

**Authors:** Junjun Wu, Tiandong Leng, Lan Jing, Nan Jiang, Daijie Chen, Youjia Hu, Zhi-Gang Xiong, Xiang-ming Zha

**Affiliations:** Department of Physiology and Cell Biology, University of South Alabama College of Medicine, 5851 USA Dr N, MSB3074, Mobile, AL 36688 USA; China State Institute of Pharmaceutical Industry, 285 Gebaini Road, Shanghai, 201203 China; Department of Neurobiology, Morehouse School of Medicine, 720 Westview Drive SW, Atlanta, 30310 GA USA; State Key Lab of New Drug & Pharmaceutical Process, Shanghai Institute of Pharmaceutical Industry, 1320 West Beijing Rd, Shanghai, 200040 China; Shanghai University School of Life Sciences, Shanghai, China

**Keywords:** ASIC, ASIC2a, Di-leucine (LL) motif, Surface expression, Trafficking

## Abstract

**Background:**

Acid-sensing ion channels (ASICs) are proton-gated cation channels that mediate acid-induced responses in neurons. ASICs are important for mechanosensation, learning and memory, fear, pain, and neuronal injury. ASIC2a is widely expressed in the nervous system and modulates ASIC channel trafficking and activity in both central and peripheral systems. Here, to better understand mechanisms regulating ASIC2a, we searched for potential protein motifs that regulate ASIC2a trafficking.

**Results and conclusions:**

We identified a LLDLL sequence in the C-terminal juxtamembrane region of ASIC2a. Deleting or mutating the LLDLL sequence increased total expression and surface levels of ASIC2a in CHO cells. Mutating either of the two LL motifs had a similar effect. We further assessed ASIC2a localization in organotypic hippocampal slice neurons. The LL motif mutants exhibited increased dendritic trafficking and elevated targeting to dendritic spines. Consistent with an efficient trafficking, the LL motif mutants increased acid-activated current density. In addition, mutating the second LL motif increased pH sensitivity of the channel. These data identify the LL motifs as a negative regulator of ASIC2a trafficking and function, and suggest novel regulatory mechanisms in acid signaling.

## Background

Acid-sensing ion channels (ASICs) are the main class of proton receptors in brain neurons. Previous studies have shown that ASICs are important for neuron physiology and synaptic plasticity, and play critical roles in fear- and anxiety-related behavior in mice [1, 2]. In addition, ASICs mediated neuronal injury in several common neurological diseases, including ischemia, multiple sclerosis, traumatic brain injury, and pain [3–10]. These results indicate that understanding the biology of ASICs is important for us to better interpret how ASICs contribute to brain function and disease.

The major ASIC subunits expressed in the brain are ASIC1a, 2a and 2b [11]. ASIC1a is the key subunit determining acid-activated current in brain neurons. In contrast, ASIC2a homomeric channels do not start to open until ~ pH 5.5. However, ASIC2a plays important modulatory roles in acid-induced responses. ASIC1a/2a heteromers show distinct current properties. Compared to ASIC1a homomers, 1a/2a heteromers have a lower pH_50_ and faster rate of desensitization [12]. In the brain, ASIC2a co-immunoprecipitated with ASIC1a and facilitated synaptic targeting of ASIC1a [13]. Deleting the *ASIC2* gene altered acid-activated current properties in CNS neurons, and reduced acid-activated calcium rise in hippocampal slices [12–15]. Consistent with these results, ASIC2 null and ASIC1a null mice exhibited similar changes in fear- and anxiety-related behavior [16]. In addition, we recently showed that deleting ASIC2 led to a region-specific protective effect against acidosis- and ischemia-induced brain injury [10].

The current data showed that ASIC2 is important in regulating the outcome of acid signaling in the brain. Moreover, ASIC2a contributed to a large proportion of functional ASICs (primarily in the form of ASIC1a/2a hetermoers) in the brain [17]. These data underline the importance for studying basic mechanisms regulating ASIC2a expression, trafficking and function. For this reason, we attempted here to identify protein motifs that regulate the expression and function of ASIC2a.

## Results and discussion

**Fig. 1:**
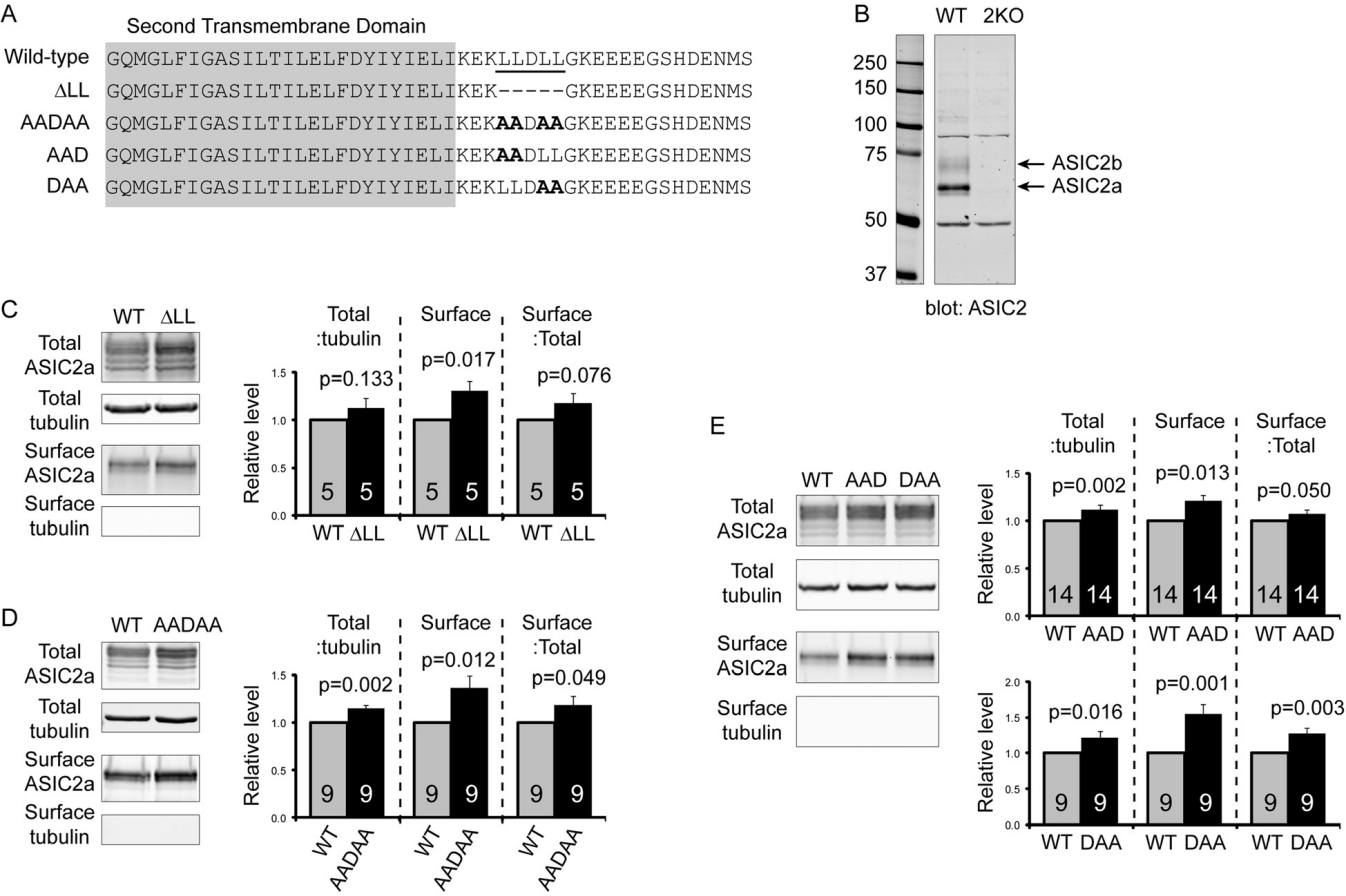
Identification of LL motifs which regulate ASIC2a expression and surface trafficking. **a** Diagram showing the C-terminus of WT ASIC2a and the mutants generated. The second transmembrane domain is highlighted by *gray shading*. **b** Blots showing the specificity of the ASIC2 antibody. WT and ASIC2−/− (2KO) brain lysate was blotted with the ASIC2 antibody. Note that the specificity of this antibody has been verified in a recent study [17] but we presented here a similar blot for the clarity of this study. **c**-**e** Representative western blot and quantification showing the effect of the corresponding mutants on expression and surface trafficking of ASIC2a. CHO cells were transfected with wild-type or mutant ASIC2a as indicated. Surface proteins were labeled by surface biotinylation and isolated by NeutrAvidin pulldown. Surface and total proteins were analyzed by Western blot. Numbers on the bars indicate the total number of repeats. *p* values are from one-tailed *t*-test

To identify potential motifs in ASIC2a, we examined the intracellular tail of ASIC2a. We noticed the presence of “_462_LLDLL” in the C-terminal juxtamembrane region. Previous studies have shown that the di-leucine (LL) motif is important for ER retention, intracellular sorting, and/or surface expression of multiple proteins [18–24]. Therefore, we hypothesized that these five amino acids are important for posttranslational processing and/or trafficking of ASIC2a. We first generated two ASIC2a mutants by deleting the LLDLL sequence (ΔLL) or mutating it to AADAA (Fig. 1a). We transfected Chinese hamster ovary (CHO)-K1 cells with WT or mutant ASIC2a, performed surface biotinylation, and blotted total proteins and surface fraction with an ASIC2 specific antibody. We have previously verified the specificity of this antibody using WT and ASIC2−/− brain [17], but presented here a similar blot for clarity (Fig. 1b). Both the ΔLL and AADAA mutant showed increased surface levels (Fig. 1c, d). This increase in surface level was associated with a 13–14 % increase in total expression and a 13–15 % increase in surface:total ratio. Next, we mutated the two LL motifs separately and generated the AAD and DAA mutants (see Fig. 1a). Mutating either LL motif increased both the expression and surface levels of ASIC2a (Fig. 1e). The increase in surface:total ratio of the AAD mutant is small (7 %) and marginal (*p* = 0.0504). In contrast, the DAA mutant exhibited a bigger effect, and led to a 27 % increase (*p* = 0.003) in surface:total ratio of ASIC2a. These data suggest that the second LL motif is more important for ASIC2a surface trafficking.

**Fig. 2:**
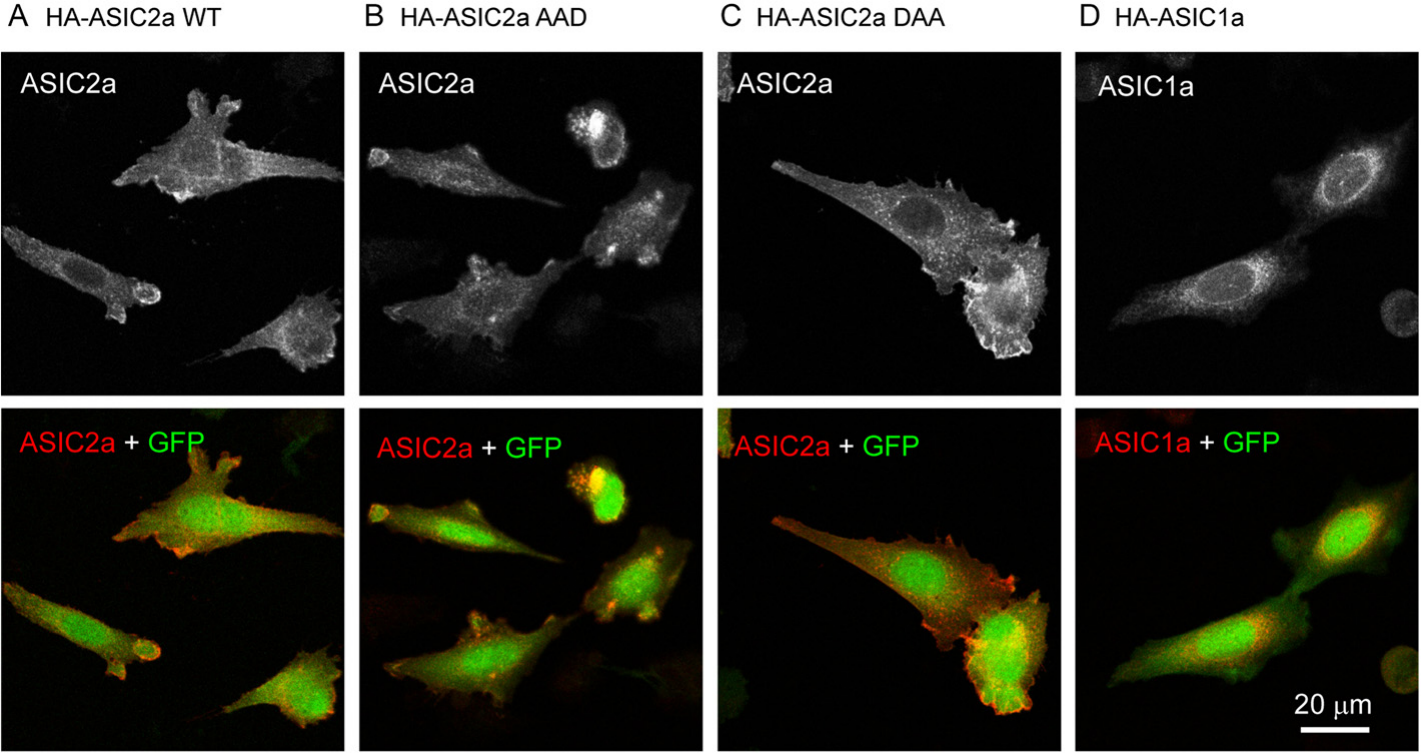
The effect of LL mutants on subcellular localization in CHO cells. CHO cells were transfected with HA-ASIC2a WT (**a**), AAD (**b**) DAA (**c**) or HA-ASIC1a (**d**) together with eGFP. ASIC localization was revealed by anti-HA immunofluorescence and visualized with confocal microscopy. Note that compared to ASIC1a (D), ASIC2a shows a more membranous localization

Next, we performed immunofluorescence in transfected CHO cells to visualize the localization of WT and mutant ASIC2a. Compared to the soluble GFP, WT ASIC2a showed a membranous localization as expected (Fig. 2a). AAD and DAA mutants exhibited a similar distribution pattern (Fig. 2b, c), although it is technically challenging to accurately quantify surface levels with immunofluorescence. We also compared the distribution of ASIC2a with that of ASIC1a. Mouse ASIC1a showed a higher intracellular staining pattern as compared to that of ASIC2a (Fig. 2d). The localization pattern of ASIC1a and ASIC2a is consistent with previous reports [17, 25].

**Fig. 3:**
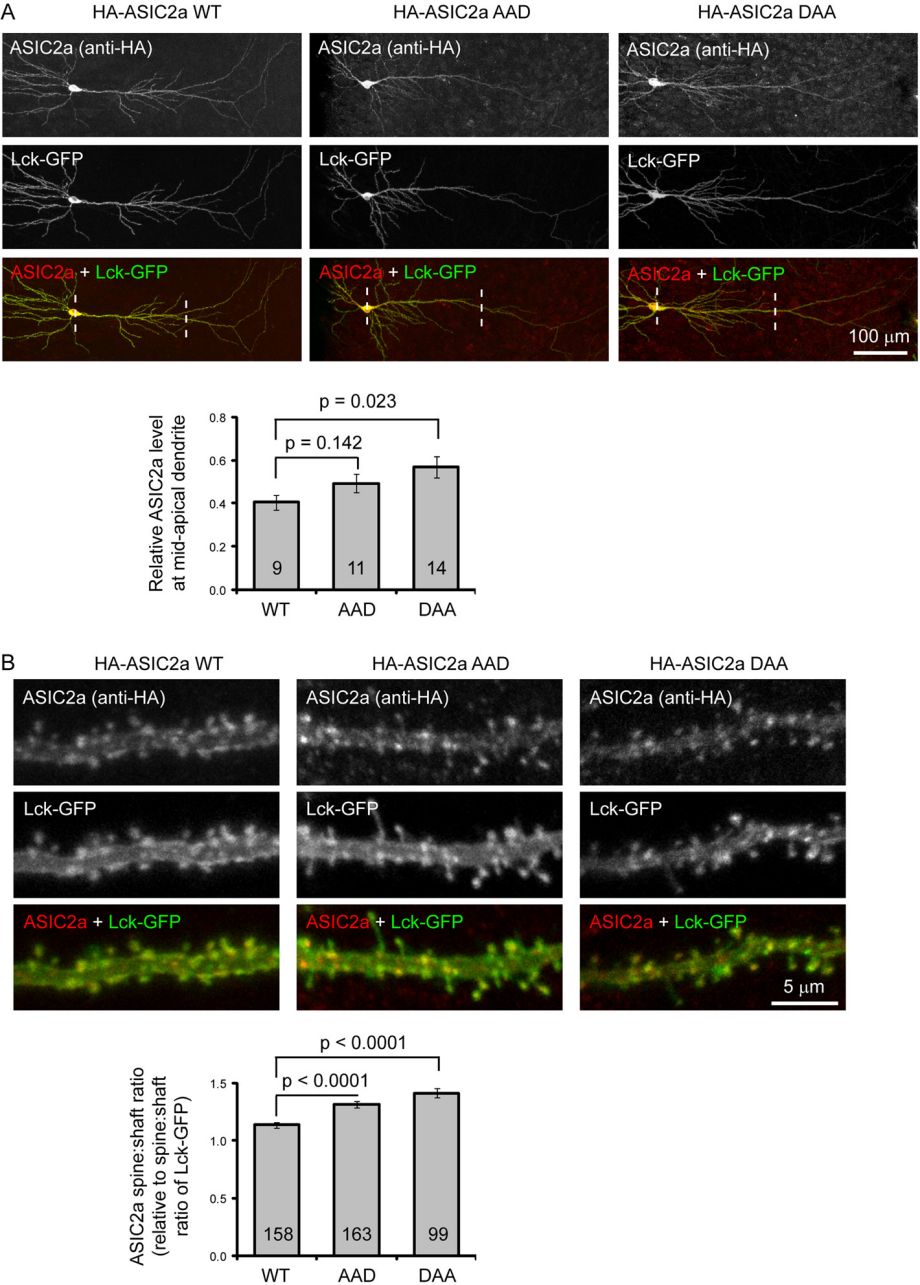
AAD and DAA mutants exhibit increased dendritic trafficking. Organotypic ASIC2−/− hippocampal slices were transfected with HA-tagged ASIC2a WT, AAD, or DAA mutant together with a membrane-targeted Lck-GFP. Localization of ASIC2a was detected using an anti-HA antibody. **a** Top: Representative images showing the overall distribution of ASIC2a in hippocampal pyrmaidal neurons. Bottom: Quantification of ASIC2a dendrite:cell body ratio. Two lines illustrate the position used for quantification: at mid-apical dendrite and cell body. The ratio is calculated as: (dendritic ASIC2a/cell body ASIC2a)/(dendritic GFP/cell body GFP). The cell body level calibrates for changes in expression while the ratio of GFP calibrates for changes in volume. *p* values were from one way ANOVA. **b**. Top: High magnification images showing ASIC2a localization in segments of mid-apical dendrites. Bottom: Quantification of ASIC2a spine:shaft ratio. To calibrate for changes in expression and volume, for a given spine, the ratio is calculated as: (spine head ASIC2a/shaft ASIC2a)/(spine head GFP/shaft GFP). *p* values were from ANOVA followed by Turkey’s HSD test. N on the bars represent total number of neurons (**a**) or spines (**b**) quantified

In a previous study, we have shown that ASIC2a exhibited a somatodendritic distribution and was enriched in dendritic spines [13]. The increase in surface trafficking of the LL motif mutants raised a question of whether these LL motifs regulate dendritic trafficking of ASIC2a. To assess dendritic targeting, we used organotypic hippocampal slices. To eliminate the potential interference from endogenous ASIC2, we cultured hippocampal slices obtained from the *ASIC2−/−* mice [26]. We transfected the slices with HA-tagged ASIC2a-WT, −AAD, or -DAA together with a membrane-targeted Lck-GFP, which facilitates the identification of transfected neurons. Similar to our previous findings using WT slices [13], ASIC2a was detected in soma and dendrites (Fig. 3a). To determine the relative trafficking into dendrities, we quantified the ratio of ASIC2a at mid-apical dendrite to that at the cell body (to control for changes in expression from neuron to neuron), and then normalized the dendrite:cell body ratio of ASIC2a to that of Lck-GFP (to control for changes in diameter or volume of the dendritic branch and/or cell body). The results showed that the DAA mutant had a significant increase in dendrite:cell body ratio as compared to WT-ASIC2a (Fig. 3a). The AAD mutant had a similar trend of increase. Next, we quantified the relative enrichment of ASIC2a in dendritic spines (Fig. 3b). The spine:shaft ratio of WT-ASIC2a, after normalizing to that of Lck-GFP, was 1.14. The normalized spine:shaft ratio for the AAD and DAA mutants were increased to 1.32 and 1.41, respectively. Both were significantly higher than that of WT (*p* < 0.01, ANOVA followed by Turkey’s HSD test). These results indicate that mutating the LL motifs led to increased dendritic trafficking and spine targeting of ASIC2a.

**Fig. 4:**
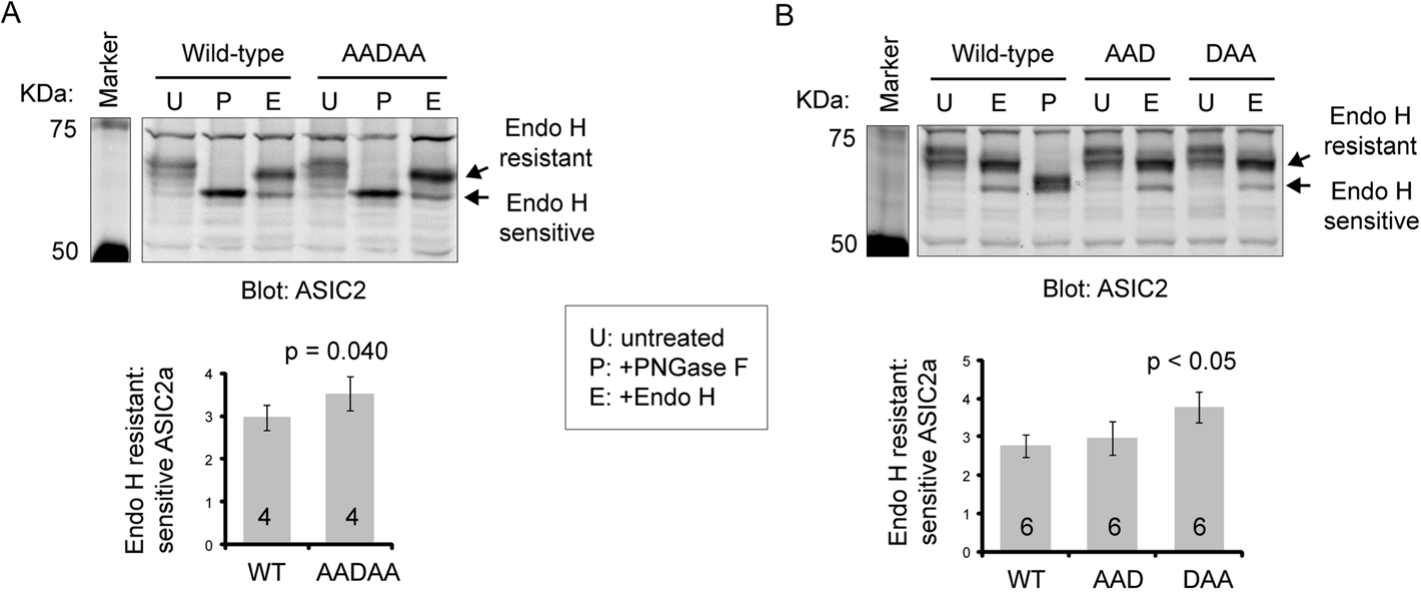
The effect of LL mutants on *N*-glycosylation of ASIC2a. Representative Western blots showing the effect of AADAA (**a**), AAD and DAA (**b**) on *N*-glycosylation status of ASIC2a. CHO cells were transfected with wild-type or mutant ASIC2a as indicated. Total cell lysates were untreated (U), treated with PNGase F (P), or treated with Endo H (E), and analyzed by Western blot. Numbers on the bar indicate total number of repeats. The *p* value was from paired *t*-test in (**a**) and Anova followed by Turkey’s HSD test in (**b**)

In our previous studies, we found that the *N*-glycosylation of ASIC1a and ASIC2a was important for their trafficking [10, 27]. We speculated that the effect of the LL motifs in ASIC2a may be due to its effect on *N*-glycosylation. To test this hypothesis, we treated CHO cell lysates with two endoglycosidases: Endo H and PNGase F. PNGase F removes all *N*-linked glycans. In contrast, Endo H only removes the core-glycans added in endoplasmic reticulum (ER) but cannot cleave more complex (“mature”) glycans that have been modified in mid- to late-Golgi. The removal of *N*-linked glycans resulted in a faster migrating population on the gel (Fig. 4). A higher proportion of Endo-H resistant fraction indicates a more efficient processing of *N*-linked glycans on the protein. As shown in Fig. 4, the ratio of Endo H resistant: sensitive population was about 2.7:1 for wild-type ASIC2a. AAD had no significant effect while both AADAA and DAA increased the proportion of Endo H-resistant ASIC2a. These results suggest that a more efficient *N*-glycosylation process may in part contribute to the increased surface expression of the AADAA and DAA mutants.

**Fig. 5:**
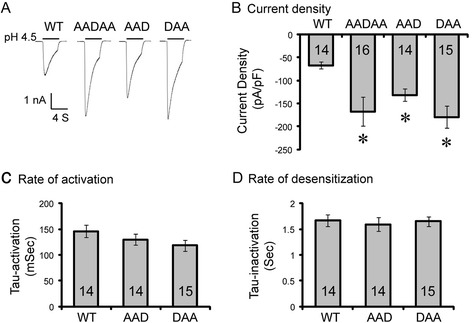
Mutating the LL motifs increases acid-activated currents. CHO cells were transfected with wild-type (WT) and mutant ASIC2a as indicated. **a**–**c** Representative traces (**a**) and quantification of pH 4.5-actived current density (**b**), rate of activation (**d**), and rate of desensitization (**d**) for ASIC2a WT and mutants. N on the bars indicate total number of cells quantified. *Astrerisks* indicate significant differences from WT (*p* < 0.05, Kruskal-Wallis One Way Analysis of Variance on Ranks followed by Dunnett’s test)

**Fig. 6:**
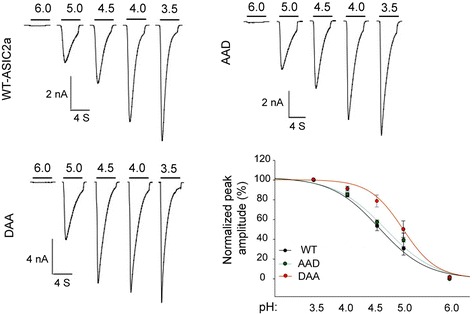
The DAA mutant shows increased pH sensitivity. Representative traces showing acid-activated current of ASIC2a WT, AAD and DAA at different stimulating pH. The plot shows pH sensitivity for the three constructs. pH_50_ for WT, AAD, and DAA was 4.53 ± 0.07, 4.64 ± 0.15, and 4.99 ± 0.07, respectively. The difference between WT and DAA was significant (*p* < 0.05). *N* = 5–6 per construct

The increase in surface ASIC2a level suggests an increase in acid-activated current. In addition, besides regulating channel biogenesis and trafficking, protein motifs may also alter channel function. To address these issues, we studied acid-activated current of CHO cells transfected with WT or mutant ASIC2a. Cells expressing either WT or mutant ASIC2a exhibited typical ASIC-type current in response to pH 4.5 stimulation (Fig. 5). This result indicates that the LL motifs are not required for ASIC2a channel to function. However, all three mutants, AADAA, AAD and DAA, exhibited significantly increased acid-activated current. This result was consistent with the biochemical data showing an increased surface levels of the mutants. Next, we asked whether channel properties are altered in the AAD and DAA mutant. The rates of activation and desensitization were not different between WT, AAD and DAA (Fig. 5c, d). Further, we studied pH sensitivity of WT, AAD and DAA mutants (Fig. 6). The AAD mutant showed similar pH_50_ as compared to the WT. In contrast, pH_50_ of the DAA mutant was increased significantly (4.99 ± 0.07 in DAA vs 4.53 ± 0.07 in WT, *p* < 0.05). One general technical consideration for most patch clamp studies is that it is harder to obtain good space clamp with large currents (e.g., in nA range). Although this may affect the exact values of current amplitude and pH_50_, it does not alter our main conclusion that the mutants had increased acid-activated current and the DAA mutant showed an increased pH sensitivity.

Previous studies have identified a number of mutants and/or motifs that affect ASIC channel trafficking and/or function [28–34]. Most of these studies focused on ASIC1a. Our results demonstrated that the LL motifs in ASIC2a are important for its trafficking and function. All the mutants that we studied here had increased surface level (Fig. 1). However, only the DAA mutant exhibited a significant increase in surface:total ratio while the AADAA mutant had a marginal effect (*p* = 0.049). These data, together with our current recordings, indicate that most of the effect on ASIC2a surface trafficking and channel function was mainly mediated by the second LL motif. We speculate that the exact location of the LL motif may contribute to the differences observed between mutating the two LL motifs. It remains unclear as to the exact mechanism of how the LL motifs regulate ASIC2a. Our data here showed that the AADAA and DAA mutants increased the maturation of *N*-linked glycans. *N*-glycosylation is an important process in protein sorting and trafficking [35]. We and others have shown previously that the maturation of *N*-linked glycans regulated ASIC1a trafficking [27, 36]. These data suggest that mutating the second LL motif facilitates posttranslational processing/sorting of ASIC2a. This speculation is consistent with previous studies showing that the LL motifs contribute to ER retention and/or sorting through trans-Golgi network [37–39]. Besides intracellular sorting, LL motifs were involved in endocytosis of several receptors, including μ and δ opoid receptors and the glucose transporter GLUT-8 [24, 40]. It is possible that some of these additional mechanisms also contribute to the regulation of ASIC2a by the LL motifs.

Do LL motifs affect the response of ASIC2a to various pharmacological inhibitors or modulators of ASICs? The LLDLL sequence locates inside the cell, right after the second transmembrane domain of ASIC2a. Therefore, mutating the LL motifs is unlikely to have a direct effect on most pharmacological reagents that bind to the extracellular side of ASICs, e.g., amiloride, diminzenes, or mitTx [41–43]. Interestingly, at the similar juxtamembrane region of ASIC1a, we and others have found two K/R rich motifs, RRGK and KEAKR [33, 34]. Mutating these K/R motifs reduced ASIC1a trafficking and acid-activated current. These data suggest that the juxtamembrane region is one important regulatory site for ASIC channels. Indeed, for multiple ion channels and receptors, the juxtamembrane region is a key region mediating protein-protein interaction and modulating trafficking and/or function [44–47]. In ASIC1a, the AP-2 complex interacted with ASIC1a through the RRGK motif and led to clathrin-mediated internalization [33]. In the future, it will be interesting to identify proteins that associate and/or interact with the LL motifs in ASIC2a. These lines of information may lead to novel approaches to manipulate acid signaling in physiology and disease.

## Conclusions

In summary*,* we identified a LLDLL sequence, which contains two LL motifs, in the C-terminus of mouse ASIC2a. Both LL motifs were involved in ASIC2a expression and trafficking. Mutating either one resulted in increased ASIC2a surface trafficking and dendritic targeting, and elevated acid-activated current. Mutating the second LL motif also increased pH sensitivity of the channel. These data indicate that the LL motifs play a critical role in modulating ASIC1a/2a heteromer trafficking and function. These data suggest potential novel mechanisms on the regulation of acid-activated responses through regulating the LL motifs in ASIC2a.

## Methods

### Mice

ASIC2−/− mice on a congenic C57 background was kindly provided by Dr. Michael Welsh. Wild-type (WT) and knockout mice were maintained as described earlier [13]. Postnatal day 5–7 (P5-7) pups (either sex) were used. Animal care met National Institutes of Health standards and all procedures were approved by the Animal Care and Use Committee at University of South Alabama.

### Constructs and reagents

Wild-type mouse ASIC2a constructs (untagged and N-terminal HA-tagged) have been described previously [13, 48]. Truncations and point mutations in ASIC2a were generated with a Quickchange mutagenesis kit (Agilent Technologies). All constructs were verified by sequencing. The ASIC2 antibody was generated by immunizing rabbit with a C-terminal peptide corresponding to the last 20 amino acid of ASIC2a [17]. Other antibodies used were: mouse anti-tubulin (University of Iowa Developmental Hybridoma Bank), rat monoclonal anti-HA (Roche, Switzerland), mouse monoclonal anti-HA (Santa Cruz Biotech., Santa Cruz, CA and Syd Labs, Malden, MA), Dylight 680-, Dylight 800-, Alexa 680- and 800-conjugated secondary antibodies (Pierce, Rockford, IL; Invitrogen, CA; Li-cor, Lincoln, NE). Other reagents used: Endo H and PNGase F (New England Biolabs, Ipswich, MA); NHS-sulfo-LC-biotin and NeutrAvidin Beads (Pierce); culture media and serum (HyClone or Invitrogen); lipofectamine 2000 (Invitrogen).

### CHO cell culture, transfection and immunofluorescence

CHO-K1 cells were purchase from ATCC. CHO cell culture and lipofectamine 2000 mediated transfection were performed as described earlier [27]. For immunofluorescence, CHO cells were initially transfected with HA-tagged ASICs together with eGFP in 35 mm dishes and re-plated into 4 well chamber glass slides one day after transfection. ASIC localization in CHO cells was detected with a rat anti-HA antibody (Roche), similar to what has been described earlier [49].

### Surface biotinylation, NeutrAvidin pull-down, de-glycosylation and western blot

Surface biotinylation, NeutrAvidin pulldown, and de-glycosylation were performed similar to what was described earlier [27]. The samples were separated by 8 % or 10 % SDS-PAGE and transferred to nitrocellulose membranes. Blots were probed with various antibodies, similar to what was described previously [27]. Antibody dilutions were: rabbit anti-ASIC2 1:500–1000; monoclonal anti-HA 1:1 K-2 K; monoclonal anti-tubulin 1:30 K; donkey or goat anti-rabbit Alexa 680 1:10,000-16,000, and donkey or goat anti-mouse Dylight 800 1:10,000-16,000. For fluorescence detection, blots were scanned with an Odyssey Infrared Imaging System. Densitometry of imaged bands was performed as described earlier [27].

### Organotypic hippocampal slice culture, transfection and immunofluorescence

Organotypic hippocampal slice was isolated from postnatal day 6–7 ASIC2a−/− mice and cultured as described earlier [13, 27]. Medium was changed every 2–3 days. Transfection was performed using a Helios-gene gun. Slices were fixed 2 days after transfection. Detailed procedures for transfection, fixation and subsequent immunofluorescence have been described in detail earlier [27].

### Confocal microscopy

Confocal images were captured using a Nikon A1 laser scanning microscope. Illumination was provided by an argon (Ar, 458, 488, 514 nm lines) and a 561 diode laser. Green and red channels were imaged sequentially, using 488 nm excitation and a 525/50 emission filter and 561 nm excitation and a 595/50 emission filter, respectively. Images were captured with a 20x or a 63x PL APO water lens. Dendritic and spine images were exported and analyzed with NIH ImageJ as described previously [27].

### Electrophysiology

Whole-cell patch-clamp recordings were performed similar to what was described previously [50]. CHO cells were transfected with WT or mutant ASIC2a together with GFP, which facilitated the identification of transfected cells. A multibarrel perfusion system (SF-77B, Warner Instruments, Hamden, CT) was used for fast perfusion. Patch pipettes have the resistance of 2–4 MΩ when filled with the intracellular solution (mM): 140 CsF, 1 CaCl_2_, 10 HEPES, 11 EGTA, 2 TEA, 4 MgCl_2_, pH 7.3, adjusted with CsOH, 290–300 mOsm. Extracellular solution contained: 140 NaCl, 5.4 KCl, 20 HEPES, 10 Glucose, 2 CaCl_2_, 1 MgCl_2_, pH 7.4 or 4.5, adjusted with NaOH and HCl, 320–330 mOsm. Whole-cell currents were recorded using Axopatch 200B amplifier (Axon Instruments, Foster City, CA) and pCLAMP software. Signals were filtered at 2 kHz, and digitized at 5 Hz using Digidata 1322A (Axon Instruments). The recordings with an access resistance of less than 10 MΩ and a leak current less than 100 pA at −60 mV were included for data analysis. Extracellular acidic solution was applied for 4 s with an interval of 1 min.

### Statistical analysis

For comparing two groups, we used paired *t*-test. For multiple comparisons, we used one way ANOVA followed by Turkey’s HSD test. For electrophysiological data, we used Kruskal-Wallis One Way Analysis of Variance on Ranks followed by Dunnett’s test. Data were reported as mean ± s.e.m. for the number of samples indicated. Differences were considered significant if *p* < 0.05.

## Abbreviations

ASIC: acid-sensing ion channel
CHO: Chinese hamster ovary
KO: knockout
WT: wild-type

## References

[1] Huang Y, Jiang N, Li J, Ji YH, Xiong ZG, Zha XM (2015). Two aspects of ASIC function: Synaptic plasticity and neuronal injury. Neuropharmacology.

[2] Wemmie JA, Taugher RJ, Kreple CJ (2013). Acid-sensing ion channels in pain and disease. Nat Rev Neurosci.

[3] Friese MA, Craner MJ, Etzensperger R, Vergo S, Wemmie JA, Welsh MJ (2007). Acid-sensing ion channel-1 contributes to axonal degeneration in autoimmune inflammation of the central nervous system. Nat Med.

[4] Xiong ZG, Zhu XM, Chu XP, Minami M, Hey J, Wei WL (2004). Neuroprotection in ischemia: blocking calcium-permeable acid-sensing ion channels. Cell.

[5] Ziemann AE, Schnizler MK, Albert GW, Severson MA, Howard Iii MA, Welsh MJ (2008). Seizure termination by acidosis depends on ASIC1a. Nat Neurosci.

[6] Pignataro G, Simon RP, Xiong ZG (2007). Prolonged activation of ASIC1a and the time window for neuroprotection in cerebral ischaemia. Brain.

[7] Diochot S, Baron A, Salinas M, Douguet D, Scarzello S, Dabert-Gay AS (2012). Black mamba venom peptides target acid-sensing ion channels to abolish pain. Nature.

[8] Mazzuca M, Heurteaux C, Alloui A, Diochot S, Baron A, Voilley N (2007). A tarantula peptide against pain via ASIC1a channels and opioid mechanisms. Nat Neurosci.

[9] Yermolaieva O, Leonard AS, Schnizler MK, Abboud FM, Welsh MJ (2004). Extracellular acidosis increases neuronal cell calcium by activating acid-sensing ion channel 1a. Proc Natl Acad Sci U S A.

[10] Jiang N, Wu J, Leng T, Yang T, Zhou Y, Jiang Q et al. Region Specific Contribution of ASIC2 to Acidosis- and Ischemia-induced Neuronal Injury. J Cereb Blood Flow Metab. 2016 in press. doi:10.1177/0271678X16630558. 10.1177/0271678X16630558 PMC5381448 26861816

[11] Zha XM (2013). Acid-sensing ion channels: trafficking and synaptic function. Mol Brain.

[12] Askwith CC, Wemmie JA, Price MP, Rokhlina T, Welsh MJ (2004). Acid-sensing ion channel 2 (ASIC2) modulates ASIC1 H + −activated currents in hippocampal neurons. J Biol Chem.

[13] Zha XM, Costa V, Harding AM, Reznikov L, Benson CJ, Welsh MJ (2009). ASIC2 subunits target acid-sensing ion channels to the synapse via an association with PSD-95. J Neurosci.

[14] Harding AM, Kusama N, Hattori T, Gautam M, Benson CJ (2014). ASIC2 subunits facilitate expression at the cell surface and confer regulation by PSD-95. PLoS One.

[15] Chiang PH, Chien TC, Chen CC, Yanagawa Y, Lien CC (2015). ASIC-dependent LTP at multiple glutamatergic synapses in amygdala network is required for fear memory. Sci. Rep..

[16] Price MP, Gong H, Parsons MG, Kundert JR, Reznikov LR, Bernardinelli L (2014). Localization and behaviors in null mice suggest that ASIC1 and ASIC2 modulate responses to aversive stimuli. Genes Brain Behav.

[17] Wu J, Xu Y, Jiang YQ, Xu J, Hu Y, Zha XM (2016). ASIC subunit ratio and differential surface trafficking in the brain. Mol. Brain.

[18] Peden AA, Park GY, Scheller RH (2001). The Di-leucine motif of vesicle-associated membrane protein 4 is required for its localization and AP-1 binding. J Biol Chem.

[19] Melvin DR, Marsh BJ, Walmsley AR, James DE, Gould GW (1999). Analysis of amino and carboxy terminal GLUT-4 targeting motifs in 3 T3-L1 adipocytes using an endosomal ablation technique. Biochemistry.

[20] Restituito S, Couve A, Bawagan H, Jourdain S, Pangalos MN, Calver AR (2005). Multiple motifs regulate the trafficking of GABA(B) receptors at distinct checkpoints within the secretory pathway. Mol Cell Neurosci.

[21] Letourneur F, Klausner RD (1992). A novel di-leucine motif and a tyrosine-based motif independently mediate lysosomal targeting and endocytosis of CD3 chains. Cell.

[22] Sandoval IV, Martinez-Arca S, Valdueza J, Palacios S, Holman GD (2000). Distinct reading of different structural determinants modulates the dileucine-mediated transport steps of the lysosomal membrane protein LIMPII and the insulin-sensitive glucose transporter GLUT4. J Biol Chem.

[23] Mangravite LM, Xiao G, Giacomini KM (2003). Localization of human equilibrative nucleoside transporters, hENT1 and hENT2, in renal epithelial cells. Am J Physiol Renal Physiol.

[24] Schmidt U, Briese S, Leicht K, Schurmann A, Joost HG, Al-Hasani H (2006). Endocytosis of the glucose transporter GLUT8 is mediated by interaction of a dileucine motif with the beta2-adaptin subunit of the AP-2 adaptor complex. J Cell Sci.

[25] Chai S, Li M, Branigan D, Xiong ZG, Simon RP (2010). Activation of acid-sensing ion channel 1a (ASIC1a) by surface trafficking. J Biol Chem.

[26] Price MP, Lewin GR, McIlwrath SL, Cheng C, Xie J, Heppenstall PA (2000). The mammalian sodium channel BNC1 is required for normal touch sensation. Nature.

[27] Jing L, Chu XP, Jiang YQ, Collier DM, Wang B, Jiang Q (2012). N-glycosylation of acid-sensing ion channel 1a regulates its trafficking and acidosis-induced spine remodeling. J Neurosci.

[28] Chen X, Kalbacher H, Grunder S (2006). Interaction of acid-sensing ion channel (ASIC) 1 with the tarantula toxin psalmotoxin 1 is state dependent. J Gen Physiol.

[29] Chen X, Polleichtner G, Kadurin I, Grunder S (2007). Zebrafish acid-sensing ion channel (ASIC) 4, characterization of homo- and heteromeric channels, and identification of regions important for activation by H+. J Biol Chem.

[30] Schnizler MK, Schnizler K, Zha XM, Hall DD, Wemmie JA, Hell JW (2009). The cytoskeletal protein alpha-actinin regulates acid-sensing ion channel 1a through a C-terminal interaction. J Biol Chem.

[31] Petroff EY, Price MP, Snitsarev V, Gong H, Korovkina V, Abboud FM (2008). Acid-sensing ion channels interact with and inhibit BK K+ channels. Proc Natl Acad Sci U S A.

[32] Yang H, Yu Y, Li WG, Yu F, Cao H, Xu TL (2009). Inherent dynamics of the acid-sensing ion channel 1 correlates with the gating mechanism. PLoS Biol.

[33] Zeng WZ, Liu DS, Duan B, Song XL, Wang X, Wei D (2013). Molecular Mechanism of Constitutive Endocytosis of Acid-Sensing Ion Channel 1a and Its Protective Function in Acidosis-Induced Neuronal Death. J Neurosci.

[34] Jing L, Chu XP, Zha XM (2013). Three distinct motifs within the C-terminus of acid-sensing ion channel 1a regulate its surface trafficking. Neuroscience.

[35] Ohtsubo K, Marth JD (2006). Glycosylation in cellular mechanisms of health and disease. Cell.

[36] Kadurin I, Golubovic A, Leisle L, Schindelin H, Grunder S (2008). Differential effects of N-glycans on surface expression suggest structural differences between the acid-sensing ion channel (ASIC) 1a and ASIC1b. Biochem J.

[37] Tikkanen R, Obermuller S, Denzer K, Pungitore R, Geuze HJ, von Figura K (2000). The dileucine motif within the tail of MPR46 is required for sorting of the receptor in endosomes. Traffic.

[38] Huan C, Greene KS, Shui B, Spizz G, Sun H, Doran RM (2008). mCLCA4 ER processing and secretion requires luminal sorting motifs. Am J Physiol Cell Physiol.

[39] Boucher R, Larkin H, Brodeur J, Gagnon H, Theriault C, Lavoie C (2008). Intracellular trafficking of LRP9 is dependent on two acidic cluster/dileucine motifs. Histochem Cell Biol.

[40] Wang W, Loh HH, Law PY (2003). The intracellular trafficking of opioid receptors directed by carboxyl tail and a di-leucine motif in Neuro2A cells. J Biol Chem.

[41] Bohlen CJ, Chesler AT, Sharif-Naeini R, Medzihradszky KF, Zhou S, King D (2011). A heteromeric Texas coral snake toxin targets acid-sensing ion channels to produce pain. Nature.

[42] Waldmann R, Champigny G, Bassilana F, Heurteaux C, Lazdunski M (1997). A proton-gated cation channel involved in acid-sensing. Nature.

[43] Chen X, Qiu L, Li M, Durrnagel S, Orser BA, Xiong ZG (2010). Diarylamidines: high potency inhibitors of acid-sensing ion channels. Neuropharmacology.

[44] Choowongkomon K, Carlin CR, Sonnichsen FD (2005). A structural model for the membrane-bound form of the juxtamembrane domain of the epidermal growth factor receptor. J Biol Chem.

[45] Ciampa EJ, Welch RC, Vanoye CG, George AL (2011). KCNE4 juxtamembrane region is required for interaction with calmodulin and for functional suppression of KCNQ1. J Biol Chem.

[46] Mohapatra DP, Siino DF, Trimmer JS (2008). Interdomain cytoplasmic interactions govern the intracellular trafficking, gating, and modulation of the Kv2.1 channel. J Neurosci.

[47] Yano H, Lee FS, Kong H, Chuang J, Arevalo J, Perez P (2001). Association of Trk neurotrophin receptors with components of the cytoplasmic dynein motor. J Neurosci.

[48] Jing L, Jiang YQ, Jiang Q, Wang B, Chu XP, Zha XM (2011). Interaction between the First Transmembrane Domain and the Thumb of Acid-sensing Ion Channel 1a is Critical for its *N-*Glycosylation and Trafficking. PLoS One.

[49] Jin W, Shen C, Jing L, Zha XM, Xia J (2010). PICK1 regulates the trafficking of ASIC1a and acidotoxicity in a BAR domain lipid binding-dependent manner. Mol Brain.

[50] Leng T, Lin J, Cottrell JE, Xiong ZG (2013). Subunit and frequency-dependent inhibition of acid sensing ion channels by local anesthetic tetracaine. Mol Pain.

